# Virulence of Aerial Conidia of* Beauveria bassiana* Produced under LED Light to* Ctenocephalides felis* (Cat Flea)

**DOI:** 10.1155/2018/1806830

**Published:** 2018-11-01

**Authors:** Sarayut Pittarate, Malee Thungrabeab, Supamit Mekchay, Patcharin Krutmuang

**Affiliations:** ^1^Department of Entomology and Plant Pathology, Faculty of Agriculture, Chiang Mai University, Chiang Mai 50200, Thailand; ^2^Agricultural Technology Research Institute, Rajamangala University of Technology Lanna, Lampang 52000, Thailand; ^3^Department of Animal and Aquatic Sciences, Faculty of Agriculture, Chiang Mai University, Chiang Mai 50200, Thailand

## Abstract

*Ctenocephalides felis *is an ectoparasitic flea species commonly found on dogs and cats. The current study verified the* in vitro *virulence of conidia of the entomopathogenic fungus* Beauveria bassiana *produced under different color LED light (red, blue, purple, green, yellow, and white) to adults of* C. felis*. The fungal isolates were cultivated on malt extract agar (MEA). Bioassay treatments used aerial conidia in test tubes. Adult fleas were obtained from a house cat in Chiang Mai province, Thailand. The experiments were composed of one control and eleven treatment groups. All of the treatments with* B. bassiana* conidia caused adult mortality after an exposure of 12 h. Among the conditions used in this study,* B. bassiana* cultured under red LED and fluorescent light were the most effective in causing mortality (100 %) in adult fleas after 36 h. The experimental results indicate that these aerial conidia of* B. bassiana* have promising potential for use in control of* C. felis* adult stages.

## 1. Introduction

The dog and cat fleas (Insecta: Siphonaptera),* Ctenocephalides canis* (Curtis), and* Ctenocephalides felis felis* (Bouché) are small, laterally flattened, wingless, and highly specialized insects. They are very important obligate hematophagous ectoparasites and have worldwide distribution [1, 2]. The adult flea lives on the host and the female lays eggs which fall from the animal and develop into larvae in the environment [3–5]. Both adult males and females are blood feeding and cause the immunoallergic skin affliction which is responsible for flea allergy dermatitis (FAD) of mammals and birds [6, 7]. Fleas can also be competent vectors for numerous pathogens, including zoonotic such as* Rickettsia typhi* (murine typhus),* Bartonella henselae* (“cat scratch disease” in humans), and* Yersinia pestis* (plague) and act as the intermediate host of the tapeworm species* Dipylidium caninum* [5, 8–10]. Traditional management of adult flea infestations on animals, during the past 15 y is by topical and oral applications of insecticides such as fipronil, imidacloprid, selamectin, and most recently by insect growth regulators (IGRs) such as lufenuron [2, 9, 11–16]. Recent studies show that these therapies eliminate the need to treat indoor and outdoor environments, and their use markedly reduces the severity and prevalence of flea infestations [17–20]. However, control of fleas by synthetic chemicals at present is very deleterious to the environment, pets, and pet owners.

Entomopathogenic fungi are microorganisms in nature that have high potential to infect, kill, control, and prevent subsequent reinfestation of many insect pests [4, 10, 21].* Beauveria bassiana* (Balsamo) Vuillemin (Hypocreales: Clavicipitaceae) is one of the most important biological control agents used in insect IPM programs and has been formulated as many commercial products. Moreover, this fungus has a wide insect-host range and has been one of the most widely studied [22, 23].* B. bassiana* produces conidia for dispersal, transmission, and infection of other insect pests. This approach is safe for the environment pets and pet owners. The objective of this study was to evaluate the virulence of aerial conidia of* B. bassiana* produced under different colors of LED light to adult cat fleas.

## 2. Materials and Methods

### 2.1. Fungus Strain and Growth Conditions

*B. bassiana *isolate BCMU4 was obtained from the Rajamangala University of Technology Lanna Collection of Entomopathogenic Fungal Cultures, Lampang. BCMU4 was originally isolated from* Nilaparvata lugens* (St*å*l) (Brown plant hopper) [Hemiptera: Delphacidae] in Pitsanulok, Thailand. Stock cultures were maintained in test tubes on slants of MEA medium (malt extract 4% soybean peptone 1% and agar 1.5%) at 25±1°C with a photoperiod of 12:12h (dark  :  light).* B. bassiana* was screened for stimulation of conidiation with six colors of LED light (red, blue, purple, green, yellow, white) and under fluorescent light and darkness. After 1 h exposure to the light treatments, cultures were kept in the dark at 27 ± 1°C and relative humidity (RH) ≥ 80% for 15 d. This study used aerial conidia produced by transferring one plug (5 mm. diam.) from the growing edge of a colony to each test tube. Conidia were suspended in a 0.01% aqueous solution of Tween 80 and quantified by haemocytometer.

### 2.2. Flea Strain

Adult fleas used in this study were the laboratory strain that has been maintained at the Insect Pathology Laboratory, Department of Entomology and Plant Pathology, Faculty of Agriculture, Chiang Mai University, Thailand. This colony originated from a wild strain taken from a house cat in Chiang Mai area and has been maintained on cats under laboratory conditions. Fleas were removed from cats via combining and counted in a test tube for every treatment (11 tr.) which was done for 5 replications. The strain has not been pressured with any insecticides since then. Bioassay treatments using 20 live fleas per one test tube were kept at room temperature for 1-2 h and were treated with one plug (0.5 mm. diam.) of* B*.* bassiana* in each test tube, incubated at 27±1°C, and mortality was checked every 12 h. The experiment had two control groups: pure agar and* Metarhizium anisopliae*. Mean mortality time and LC_50_ were calculated and statistical analysis used one-way ANOVA and significance between treatments was tested with the LSD method (*P < *0.05).

## 3. Results

**Figure 1:**
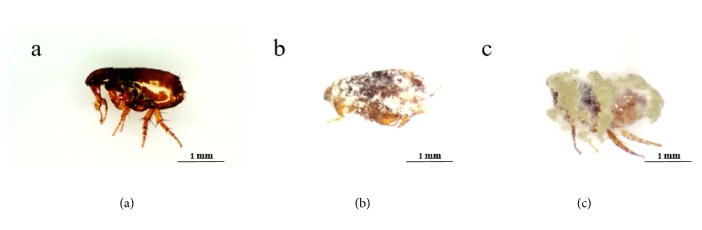
Cumulative mortalities of* Ctenocephalides* sp. male and female adults after exposure to fungal aerial conidia. (a) Healthy adult of* Ctenocephalides* sp., (b) adults of* Ctenocephalides* sp. (72 h) after death caused by* B. bassiana *isolate BCMU4, and (c) Adults of* Ctenocephalides* sp. (72 h) after death caused by* M. anisopliae* All the scales bars are 1 mm.

Previous studies have shown that different color LED light has great potential to stimulate* B. bassiana *conidial production with a high virulence to pests. In the current study, the mortality of* Ctenocephalides* sp. observed in treatments 1-11 was time-dependent (Table 1). Adult fleas treated with one plug (0.5 mm. diam.) of the fungus grown on MEA,* B. bassiana* + [red (0.21×10^8^ conidia/ml), blue (0.03×10^8^ conidia/ml), purple (0.06 ×10^8^ conidia/ml), green (0.11×10^8^ conidia/ml), yellow (0.07×10^8^ conidia/ml), white LED (0.05×10^8^ conidia/ml)],* B. bassiana* + (fluorescent (0.09×10^8^ conidia/ml), dark (0.09×10^8^ conidia/ml)), and two isolates of* M. anisopliae* (4849 (0.04×10^7^ conidia/ml), 2MG (0.13×10^8^ conidia/ml)) had significantly different mortalities at 12 h after treatment. The results revealed that the cumulative mortalities of adults at 12 h ranged between 6 and 27 percent. After 24 h, all of fungal treatments showed a cumulative flea mortality of more than 75 percent. During the same period,* B. bassiana* + (red, purple) and two isolates of* M. anisopliae* (4849, 2MG) showed the highest cumulative mortalities of 93, 89, 92, and 92%, respectively, and were not significantly different, while 100% mortality was reached in the treatments* B. bassiana* + (red, fluorescent) and* M. anisopliae* (4849) after 36 h (Table 1). All fleas treated with entomopathogenic fungi were killed within 48 h after exposure. No mortality was observed in the control group. After dead host fungal will formation mycelial outgrowth from insect cadaver and production of new conidia (Figure 1).

## 4. Discussion

To the best of our knowledge, the work of seeking efficient entomopathogenic fungi against* Ctenocephalides* sp. was also only undertaken by De-Melo et al. [1, 21, 24].* B. bassiana *has shown pathogenicity as a biological control agent for different arthropod species and it is commercially available as various mycoinsecticides [1, 10, 21]. In our study, aerial conidia of* B. bassiana* produced under LED light start killing fleas within 12 h after treatment administration [21]. Moreover,* B. bassiana* had a cumulative flea mortality of 100% in 36-48 h as short time similar some synthetic chemicals [2, 7, 9, 14, 23]. In general, for flea control in dogs and cats garden or backyard areas are treated with insecticides in an attempt to control flea infestations. However, such applications of insecticides are often poorly targeted. Hence, entomopathogenic agents have benefits in terms of effectiveness against flea infestations. It is difficult for fleas to become resistant to entomopathogenic fungi. But this insect can readily develop resistance to chemical insecticides [17, 25]. Similarly, in a study conducted by Coles and Dryden [22] it was reported that control of fleas with entomopathogenic fungi can be conducted safely in terms of positive benefits for the environment, pets, and pet owners [10, 19]. Previous research [26] showed that different isolates of* M. anisopliae* had different effects on insect pests. So, identification, application and screening of different fungal isolates in bioassays may provide promising biopesticides. Our study screened both* B. bassiana* and* M. anisopliae* with activity against adult fleas. The current research showed that growth of* B. bassiana* under LED lights enhanced production of conidia that resulted in 100% overall flea mortality. Future research will examine the ovicidal and larvicidal capacity of this entomopathogenic fungus. In the future such entomopathogenic fungi could be developed as bioinsecticides for practical use in pet care products like Chaingard® pets powder. The evidence presented here indicates that both* B. bassiana* and* M. anisopliae* have a high level of adulticidal activity, and potential for inclusion in flea management systems.

## 5. Conclusions

A previous study showed the efficacy of flea control by using aerial conidia of* B. bassiana *produced under difference colors of LED light was relatively high ranging from 78 to 100 percent and rapidly occurred from 24 to 36 h after exposure. Comparisons of conidia of* B. bassiana* produced under different colors of LED light to enhance their virulence and efficacy were obtained from the experiments. Conidia of* B. bassiana *produced under different colors of LED light caused infection which involves integument penetration at 12 h and total colonization of the insect at 48 h after exposure. Therefore, these results indicate that* B. bassiana* culture under LED light has great potential as a candidate for biological control of* Ctenocephalides* sp. adult stages. The potential of this entomopathogenic fungus for controlling fleas needs continued study, and the research should be expanded to include other ectoparasites of dogs and cats.

## Figures and Tables

**Table 1:** The ability of aerial conidia of *B. bassiana* isolate BCMU4 produced under LED lights to control *Ctenocephalides felis*.

Fungus conditions	Adult mortality (%) at infestation day			
	12 h	24 h	36 h	48 h
*B. bassiana* + red	27a^*∗*^	93a	100a	100
*B. bassiana* + blue	25a	87bc	93b	100
*B. bassiana* + purple	23a	89ab	91bc	100
*B. bassiana* + green	11bc	84cd	89cd	100
*B. bassiana* + yellow	13bc	80de	89cd	100
*B. bassiana *+ white	10cd	83cd	87d	100
*B. bassiana* + fluorescent	15b	86bc	100a	100
*B. bassiana* + dark	6d	78e	86d	100
*M. anisopliae* 4849	25a	92a	100a	100
*M. anisopliae* 2MG	26a	92a	99a	100
Control group	0e	0f	0e	0

LSD (0.05%)	2.2361	2.0207	1.7795	-

CV	23.44	4.44	3.61	-

^*∗*^Means followed by the same letter in the same row in each parameter are not significantly different by LSD (P< 0.05). Means calculated from five replications. Experiments were kept at 27±1°C and relative humidity (RH) ≥ 80%.

## Data Availability

The data used to support the findings of this study are included within the article.

## Abbreviations

LED: Light emitting diodes
MEA: Malt extract agar.
